# Supplementary material for: Quantifying Yeast Microtubules and Spindles Using the Toolkit for Automated Microtubule Tracking (TAMiT)
Source: Biomolecules. 2023 Jun 4;13(6):939. doi: 10.3390/biom13060939 (PMC10296093; doi:10.3390/biom13060939)
Supplement: Supplementary file 1 [file biomolecules-13-00939-s001.zip › biomolecules-1862527-supplementary.pdf]

Supplemental Material

1 Feature Inheritance

TAMiT implements features as instances of classes. The structure of inheritance is shown in Figure S1. The most general class is a *Feature*. There are two types of features. *Basic Elements* are physical and geometric objects that are associated with fluorescent intensity. These include lines, spots, and curves. *Organizers* structurally organize the basic elements to form composite features. An organizer can contain basic elements or other organizers. For example, an *Aster* contains one spot, and multiple lines. A *Spindle* and a *Spindle Bud* contains a line (for the spindle), and two asters (for SPB and astral microtubules).

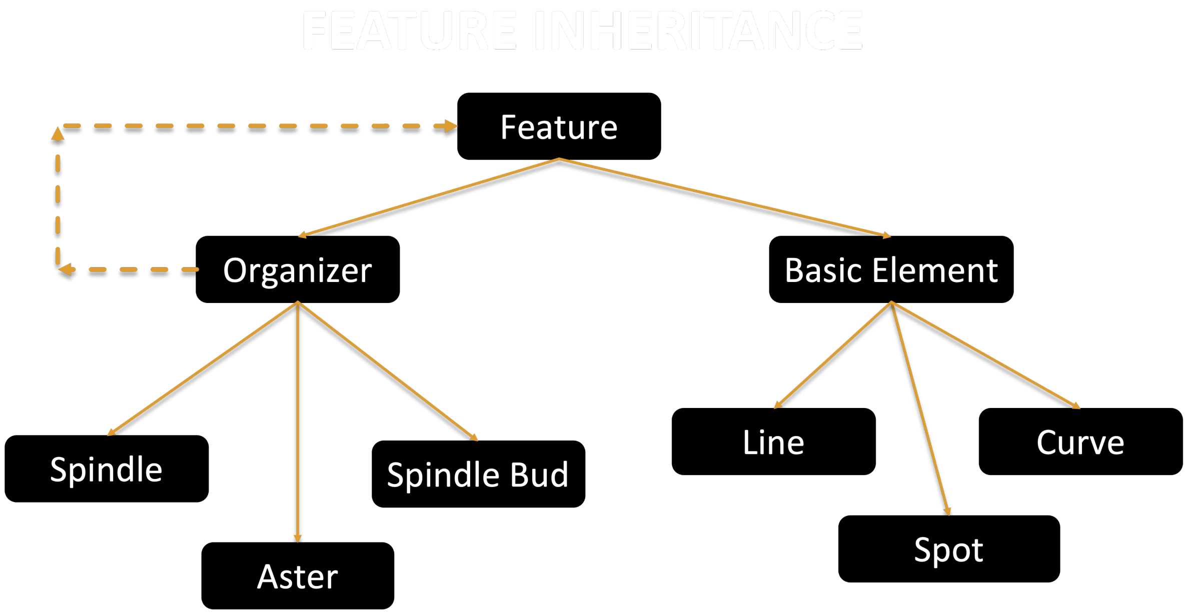

Figure S1: Inheritance in TAMiT. A feature is the most general object. There are two kinds of features, an Organizer and a Basic Element. Basic Elements are physical and geometric objects that have an intensity profile. Organizers are composed of other features and organize them in various ways. For example, the aster organizer contains a spot and multiple lines.

2 SPB detection

The detection process is independent for each frame in the image stack. For a 3D image (XYZ), we take a maximum intensity projection along Z. This gives us a 2D image, which we use for the estimation process. Here, we show the detection process for a spindle pole body (SPB) in a *S. pombe* monopolar spindle (Figure S2A). We begin with pre-processing where we apply a gaussian filter on the image (Figure S2B). This gaussian filter (standard deviation equal to 2 pixels) is used to enhance the tubulin signal. The next step is to identify the region of the SPB. We perform otsu thresholding, followed by an extended-maxima H transform to find an extended region belonging to the SPB (Figure S2C). Finally, the maximum intensity pixel within this region is used as the estimate for the SPB location

(Figure S2D). For the Z position of the SPB, we find the Z pixel, with fixed XY location, with the maximum intensity.

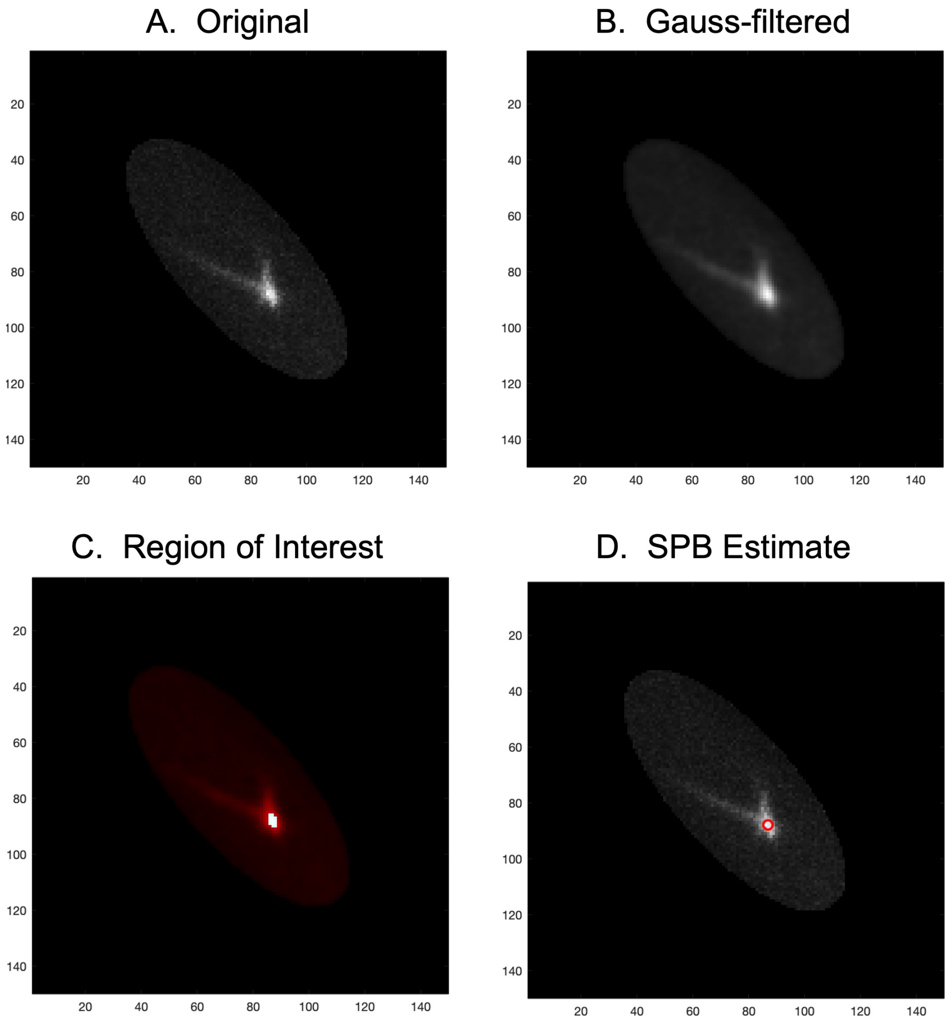

Figure S2: Spindle pole body (SPB) detection. All images are maximum intensity projections in Z of the full 3D image. (**A**) Original image showing a monopolar spindle. The SPB is the brightest region. (**B**) Gaussian-filtering with a filter of size equal to the point spread function enhances the fluorescent signal of tubulin. (**C**) Otsu thresholding followed by an extended maxima transform yields a region of interest. (**D**) SPB estimate is the maximum intensity pixel in the region of interest.

3 Straight microtubule detection

Line detection for straight microtubules starts with an estimation for a start point. This may be done using either SPB detection for a *S. pombe* monopolar spindle (Sec. 2) or spindle detection for a *S. pombe* mitotic spindle (Sec. 4). The line algorithm executes the following steps:

1. Given a start point (Figure S3A), perform an azimuthal sweep and calculate the summed intensity along different directions.
2. Find peaks in the intensity plot to identify angles with enhanced intensities (Figure S3B).
3. For each identified peak/angle, measure the intensity at different radii (Figure S3C).
4. We smooth the signal and find the radius when it falls below a threshold criterion (Figure S3C). This can be used to find the length of the line.

5. Given the start point, an angle, and a length, we find the end-point of each line.

The final estimate is shown in Figure S3D.

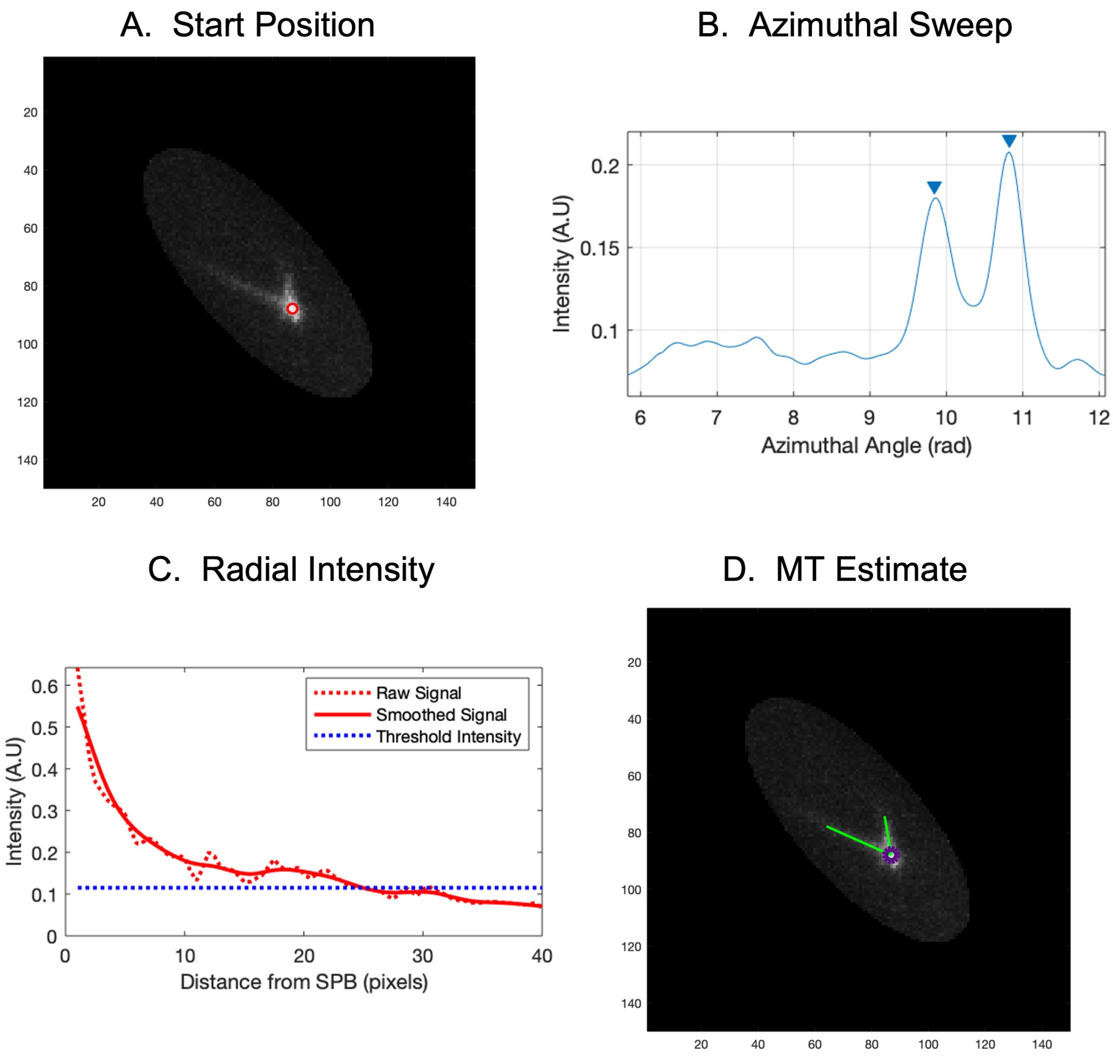

Figure S3: Detection of straight microtubules. All images are maximum intensity projections in Z of the full 3D image. (**A**) The start position where the microtubule search algorithm is initiated. This is normally the SPB location. (**B**) Azimuthal sweep gives the summed intensity at different angles. A peak finder gives two clear peaks corresponding to the two microtubules visible in the original image. (**C**) Radial intensity along the direction of one of the microtubules. A threshold determines the end-point of the microtubule along this direction. (**D**) The final estimate showing the detected microtubules in green and the SPB in purple.

4 Spindle detection

We start with a 2D maximum intensity projection in Z as mentioned earlier, and apply a gaussian filter (standard deviation of 2 pixels) (Figure S4A). Next, we use iterative thresholding to find an elongated region in the image. The thresholding process works as follows:

1. Initialize a threshold value equal to the median intensity of the image

2. Generate a boolean mask with pixels above the threshold given value 1 and below the threshold given value 0.

3. Find a convex hull (ensures that there is a single connected region in the image
4. Find the major and minor axis length of this region, and subsequently the aspect ratio.
5. If the aspect ratio is less than 4 (tunable parameter, should be set around 5 to yield a region that represents the spindle), then increase threshold by 1% and repeat steps 2-5. If condition is satisfied, or a maximum threshold value is reached, then stop iterative thresholding and accept the boolean mask (Figure S4B).

Given the mask (which may contain multiple regions), pick the region with the greatest integrated intensity. Next, we find the corresponding major axis which is expected to align with the spindle axis (Figure S4C). Measuring the intensity along this axis, we find two end points which satisfy an automated threshold criterion (Figure S4D). These end-points correspond to the SPB locations and the line connecting them represents an estimate for the spindle (Figure S4E).

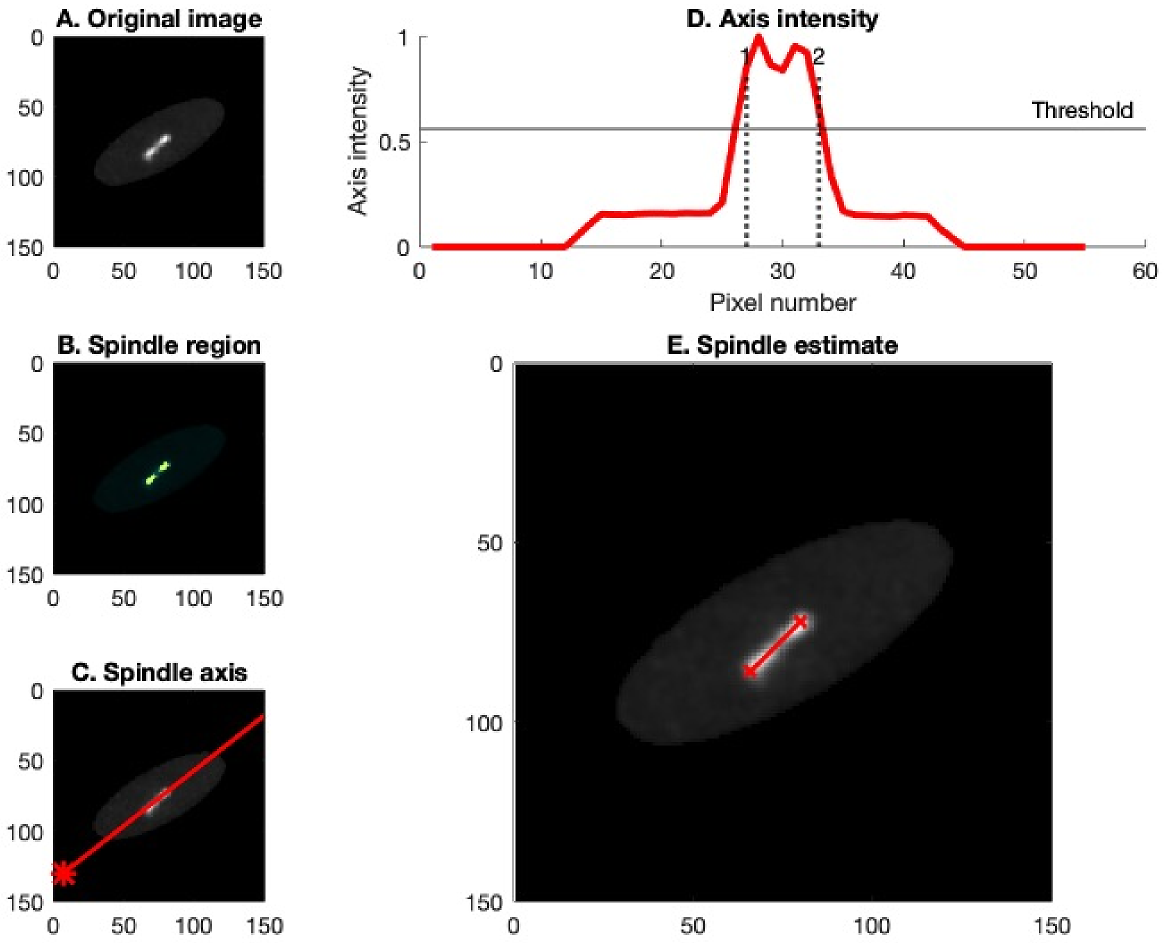

Figure S4: Spindle detection. All images are maximum intensity projections in Z of the full 3D image. (**A**) Original image showing the bright spindle. (**B**) Iterative thresholding yields regions of interest belonging to the spindle. (**C**) Axis passing through the best region of interest. (**D**) Intensity along the axis. A threshold determines the two end-points belonging to the the spindle end-points. (**E**) The spindle estimate.

5 Curved microtubule detection

Here, we show curve detection performed on a time frame from *S. cerevisiae* (Figure S5A). To find curved microtubules, we need estimated start points (Figure S5B). We begin by using steerable filtering

to find pixels showing line-like intensity (Figure S5C). This given us paths which can be identified using iterative techniques. For a start point, we being by performing an azimuthal sweep to identify the number and the initial direction of curves emanating from this point. Then, for each curve, we find its coordinates by following these steps:

1. Begin with a start point, an initial angle, and a path image (Figure S5C).
2. Find the next point along the path by azimuthally scanning and taking a small radial step in the direction of highest intensity.
3. If the maximum intensity is below a threshold value, reject this point
4. Reduce the radial step size and attempt to find another point. If intensity is still below a threshold, reject and stop finding more points along this path/curve.
5. With the new point (if found), repeat steps 2-5 until no more points are found.
6. The final coordinates are shown in Figure S5D,E.
7. Now, using the original image, reduce the length of found curves until the intensity at the end-point satisfies a threshold criterion.

The final estimate with curved microtubules is shown in Figure S5F.

6 *S. pombe* Strains

7 Movies

- Movie 1: Microtubule detection in *S. pombe* mitotic cell. Left (original MIP), center (fitted MIP), and right (2D features). Colorbar represents the position in Z (μm).
- Movie 2: Microtubule detection in *S. pombe* monopolar cell. Left (original MIP), center (fitted MIP), and right (2D features). Colorbar represents the position in Z (μm).
- Movie 3: Microtubule detection in *S. cerevisiae* mitotic cell. Left (original MIP), center (fitted MIP), and right (2D features). Colorbar represents the position in Z (μm).

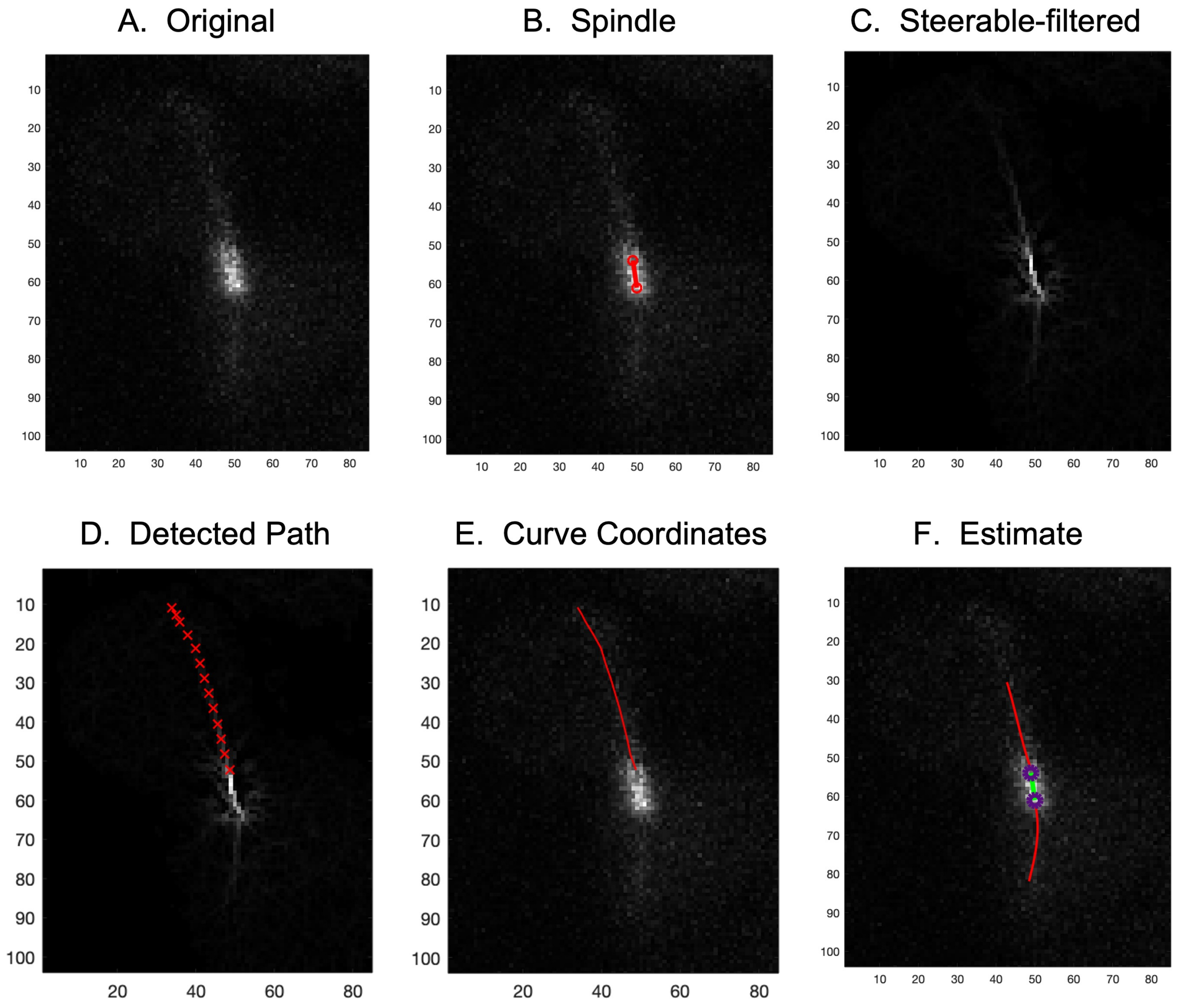

Figure S5: Detection of curved microtubules. All images are maximum intensity projections in Z of the full 3D image. (**A**) Original image showing a budding yeast metaphase cell. (**B**) The detected spindle found using the algorithm presented in Section 4. (**C**) Steerable filtering accentuates pixels where a line passes through. (**D**) Detected path of a curve (red) using a combination of a restricted azimuthal sweep, and a fixed radial step. (**E**) Curve coordinates generated from the path shown in D. (**F**) Final estimate of the curved microtubules (red) along with the spindle (green) and the SPBs (purple). Microtubule length is reduced based on intensity considerations mentioned in Section 5.

Strain #	Genotype	Source
MB850	cut7-24, Z:adh15:mCherry-atb3:natMX6, ura4-D18, h+	this study
MB857	cut7-24, Z:adh15:mCherry-atb3:natMX6, klp6Δ::ura4+, ura4-D18, h-	this study
MB863	cut7-24, Z:adh15:mCherry-atb3:natMX6, alp14Δ::Kanr, ura4-D18, h+	this study
MB890	cut7-24, Z:adh15:mCherry-atb3:natMX6, cls1-36:ura4+, ura4-D18, h+	this study
MB919	cut11-7, Z:adh15:mCherry-atb3:natMX6, mis12-GFP::leu1+, leu1-32, ura4-D18, h+	this study

Figure S6: S. pombe strain information
